# Maternal and Placental DNA Methylation Changes Associated with the Pathogenesis of Gestational Diabetes Mellitus

**DOI:** 10.3390/nu15010070

**Published:** 2022-12-23

**Authors:** Peng Xu, Shuai Dong, Linlin Wu, Yule Bai, Xueqing Bi, Yaping Li, Chang Shu

**Affiliations:** Department of Obstetrics, Obstetrics and Gynaecology Center, The First Hospital of Jilin University, Jilin University, Changchun 130021, China

**Keywords:** gestational diabetes mellitus (GDM), DNA methylation, epigenetics, insulin-like growth factor, adipokines, leptin, adiponectin, TNF-α, insulin resistance

## Abstract

Gestational diabetes mellitus (GDM) is an important metabolic complication of pregnancy, which affects the future health of both the mother and the newborn. The pathogenesis of GDM is not completely clear, but what is clear is that with the development and growth of the placenta, GDM onset and blood glucose is difficult to control, while gestational diabetes patients’ blood glucose drops and reaches normal after placenta delivery. This may be associated with placental secretion of insulin-like growth factor, adipokines, tumor necrosis factor-α, cytokines and insulin resistance. Therefore, endocrine secretion of placenta plays a key role in the pathogenesis of GDM. The influence of DNA methylation of these molecules and pathway-related genes on gene expression is also closely related to the pathogenesis of GDM. Here, this review attempts to clarify the pathogenesis of GDM and the related maternal and placental DNA methylation changes and how they affect metabolic pathways.

## 1. Introduction

Gestational diabetes mellitus (GDM) is a metabolic disease defined as abnormal glucose tolerance that occurs during pregnancy [1]. The morbidity caused by GDM varies from 9.5 to 26.6% in different regions of the world [2]. GDM can cause perinatal and neonatal complications. Perinatal complications mainly include macrosomia, shoulder dystocia, preeclampsia, polyhydramnios, fetal growth restriction, premature delivery and infection; neonatal complications include neonatal hypoglycemia, respiratory distress syndrome, etc. However, the pathogenesis of GDM has not been clarified. 

**Figure 1:**
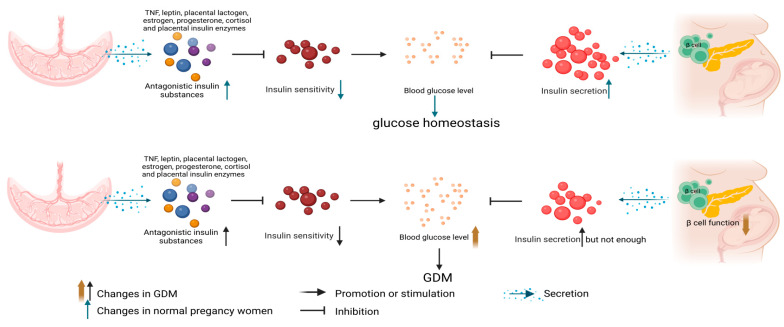
Pathogenesis of GDM. The images in this figure were taken from https://biorender.com/ (accessed on 22 November 2022).

It is widely accepted that pathogenesis of GDM may be related to the maternal inability to tolerate insulin resistance during pregnancy. In the first trimester, the glucose level is found to decrease gradually with gestational weeks largely due to the following reasons: First, the fetal demand for glucose increases; next, the increase of the glomerular filtration rate leads to the increase of glucose excretion from urine; third, the estrogen and progesterone increase maternal glucose utilization. In the second and third trimesters, the sensitivity of pregnant women to insulin decreases with the increase of the gestational age due to the placental secretion of insulin antagonists such as tumor necrosis factor (TNF), leptin, placental lactogen, estrogen, progesterone, cortisol and placental insulin. In order to maintain blood glucose homeostasis, insulin demand is increased. However, for pregnant women with insufficient islet function, the decline of β cell function and the inability to secrete more insulin for compensation will cause the onset of GDM, as shown in Figure 1. Surprisingly, most patients’ blood glucose has returned to the normal level by the Oral Glucose Tolerance Test (OGTT) two days after delivery [3], indicating that the incidence of GDM may be closely related to the placenta. The placenta is a powerful endocrine organ as well as a nutrient delivery organ for the fetus. The antagonistic insulin substances secreted by the placenta can affect the metabolic changes of the mother. Therefore, the effect of hormones secreted by the placenta on the pathogenesis of GDM have become the focus of the current research on the mechanism of GDM.

DNA methylation, one of the most common epigenetic modifications, has received considerable attention. It refers to the addition of a methyl group to the 5’ end of DNA under the action of DNA methyltransferase (DNMT), thereby affecting the regulation of gene expression. Usually, this modification occurs on cytosine (C)—phosphate (p)—guanine (G) (CpG). A section of DNA rich in CpG in the genome is called a CpG island, with a length of 300–3000 bp. CpG islands are usually located in the promoter region of the gene, so the CpG island of the gene promoter is a common location where methylation occurs. Approximately 70–80% of the CpG in the human genome is methylated [4]. There are three possible mechanisms of DNA methylation in gene expression regulation. The first is the extension of 5-methylcytosine into the large groove of DNA double helix, affecting the binding of transcription factors. The second is the binding of sequence specific methylated DNA binding proteins (MDBP-l and MDBP-2) to the methylated promoter sequence to inhibit the binding of transcription factors to target sequences. Thirdly, methylated CpG binding proteins (MeCP1 and MeCP2) bind methylated dinucleotide CpG, similar to the role of transcriptional inhibitory proteins. MeCP1 needs 12 methylated CpGs to bind methylated DNA, but MeCP2 only needs one methylated CpG [5].

In addition, macrosomia, one of the complications of GDM, is related to the Insulin like growth factor 1 (IGF-1) axis. Its expression level is higher in GDM maternal blood and umbilical cord blood than in normal pregnant women, but there is no significant difference in placenta [6,7]. This demonstrates the solitary regulation of IGF-1 in the placenta. However, many GDM patients are associated with obesity, and the levels of adipokines such as leptin and adiponectin in their bodies are very different from those of normal pregnant women. These adipokines are also involved in glycolipid metabolism, and there are unique ways of regulation in the placenta to affect the level of blood sugar. The level of inflammation in obese patients is also different from that in normal pregnant women. The mRNA and protein levels of some cytokines, such as TNF-α, in obese patients are higher than those in normal people [8]. TNF-α also has an effect on glucose metabolism, and the placenta also shows higher secretion of TNF-α under the condition of hyperglycemia. Methylation changes of corresponding genes in the metabolic pathways of the above factors have also been found, which play an important role in the regulation of GDM gene expression. DNA methylation of molecules involved in the pathogenesis of GDM has indeed been reported in previous articles, but the role of these molecules in the pathogenesis of GDM and the related pathways have not been clarified. This review attempts to elucidate the pathogenesis of GDM and the changes and functions of maternal and placental DNA methylation.

## 2. Role of IGF-1 Axis in the Pathogenesis of GDM and Methylation Changes

### 2.1. Regulation Mechanism of IGF-1

The insulin-like growth factor (IGF) includes IGF-1 and insulin-like growth factor 2 (IGF-2), which are expressed in the placenta [9]. They play an important role in cell growth in all systems. Growth hormone releasing hormone (GHRH) secreted by the hypothalamus regulates the secretion of growth hormone (GH), and GH can promote the increase in IGF-1 level. This level in maternal blood during pregnancy is higher than that during non-pregnancy, because the placenta secretes more placental growth hormone, also known as Growth Hormone Variant (GH-V), which replaces the regulatory role of pituitary GH and forms a unique feedback mechanism [10]. The placental lactogen and GH-V produced by the placenta stimulate the increase of liver cytokines, which leads to the increase in free fatty acid levels, IGF-1 and insulin levels [11]. IGF-1 and insulin levels are higher in overweight women during pregnancy [12], and obesity during pregnancy is an important risk factor for GDM. Insulin-like growth factor I receptor (IGF1R) is a tetramer receptor tyrosine kinase (RTK), which is mainly bound to IGF-1. It can activate the PI3K/AKT and MAPK pathways, further activating the expression of mTOR in the cell nucleus. The function of mTOR is related to cell survival and proliferation [13]. Glucose transporter-4 (GLUT4) is a Glucose transporter responsible for bringing glucose into cells to be used as the primary transporter for energy. In the baseline state, GLUT4 can be distributed in GLUT4 storage vesicle (GSV). When the insulin receptor signals, GSV dissociates and releases glucose outside the cell. IGF-1 and Insulin Receptor (IR) bind to the IR with low affinity [14], resulting in a phosphorylation cascade of the PI3K/AKT pathway and phosphorylation of AS160 (Akt Substrate of 160 KDa) protein. AS160 further dissociates GLUT-4 from the GLUT4 storage vesicle, resulting in an increase in the cell surface GLUT-4. This in turn increases glucose transport to glucose sensitive tissues [15,16], such as maternal liver, muscle and fat, increasing the utilization of glucose, and then regulating the blood glucose decrease. However, IGF-1 binds insulin-like growth factor binding proteins (IGFBP1-IGFBP7) [17] to form dimers or trimers. For example, after IGFBP-1 or IGFBP-2 binds to IGF-1, the binding of IGF-1 and IGF1R is affected by prolonging the half-life of IGF-1, thereby reducing the effectiveness of IGF-1 [18].

### 2.2. Role of IGF-1 in the Pathogenesis of GDM

**Figure 2:**
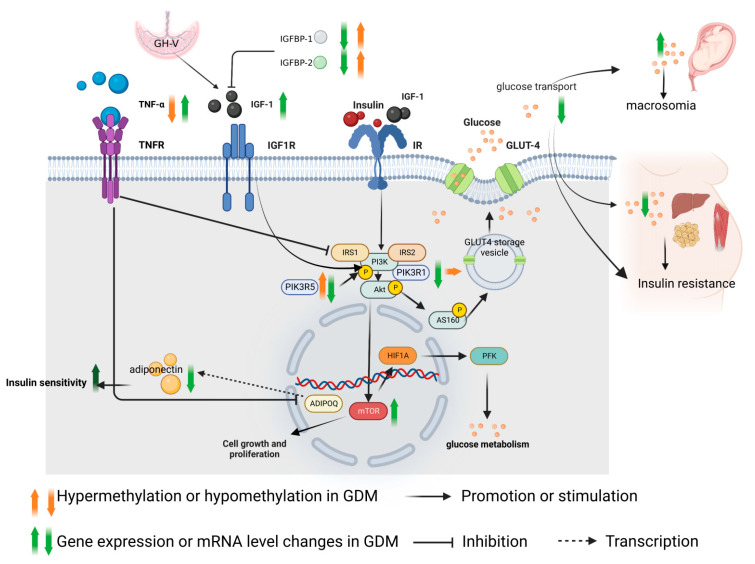
Changes of IGF-1 axis, insulin related cellular signal transduction pathway and methylation of related molecules in GDM. The images in this figure were taken from https://biorender.com/ (accessed on 22 November 2022).

It has been reported that in GDM, the IGF-1 level in maternal peripheral circulation is higher than that in normal pregnant women. Although the IGF-1 level in cord blood is higher than that in normal pregnant women, it is not more significantly increased than that in maternal blood. In addition, the IGF-1 level in maternal blood is positively correlated with the birth weight of the newborn and the glucose level from the OGTT test [6]. Although in placenta the expression of IGF-I mRNA of GDM patients is not significantly different from that in normal pregnant women, the expression of IGF1R mRNA in placenta was significantly increased [7]. In the placenta of GDM, the insulin/IGF-1 pathway is over activated, especially the downstream mTORC1 (mechanical target of rapamycin) pathway [19]. The mTORC1 target is responsible for the regulation of cell proliferation, tissue differentiation and blood glucose regulation [20]. mTORC1 increases the translation of the transcription factor. Hypoxia Inducible Factor 1 Subunit Alpha (HIF1A) promotes the expression of phosphofructose kinase (PFK) and cell growth by promoting glucose metabolism [21], as shown in Figure 2. The placenta transports this glucose to the fetus, resulting in increased nutrient transport between the mother and fetus, which promotes fetal overgrowth and results in macrosomia.

It has been reported that the promoter regions of IGFBP-1 and IGFBP-2 in the placenta of GDM patients are significantly hypermethylated. This leads to the decreased expression of IGFBP-1 and IGFBP-2 mRNA by inhibiting the binding of transcription factors to the target sequence, reducing the binding of IGF-1 and increasing the dissociation of IGF-1. Thus, the binding of IGF-1 and IGF1R is promoted, so the PI3K/AKT pathway is over activated, leading to the increased transport of glucose to the fetus; as a result, there is the occurrence of macrosomia. However, the promoter methylation of IGFBP-1, IGFBP-2 and IGFBP-6 in blood samples of GDM patients has no significant change compared with that of the control group [22]. In conclusion, although the placental IGF-1 level does not change significantly in GDM compared with that of normal pregnant women, IGFBP1 and IGFBP2 in GDM decreases and IGF1R increases, which improves the utilization efficiency of IGF-1 and leads to the overactivation of mTORC1 and the increase of glucose transport to the fetus. This also suggests that the regulation of the IGF-1 axis in placenta may be independent of maternal self-regulation (as Figure 2).

## 3. Adipokine

Obesity is an important risk factor for the onset of GDM, and the hormone secretion level of obese patients is significantly different from that of normal pregnancies, as shown in Table 1 [23,24,25]. An important class of these hormones is secreted by adipose tissue, collectively known as adipokine hormones, including leptin [26], adiponectin, resistin, chemerin, etc. Adipokines participate in a variety of metabolic processes, including insulin sensitivity, insulin secretion, appetite control, fat distribution, energy consumption, inflammation, regulation of fat formation and chemotaxis of immune cells to adipose tissue [27]. The change of adipokine secretion can lead to the change of glucose homeostasis during pregnancy, and adipokine is also associated with the pathogenesis of GDM, so adipokines have become a hot spot in GDM research.

### 3.1. Role of Leptin Axis in the Pathogenesis of GDM and Methylation Changes

#### 3.1.1. Leptin in the Pathogenesis of GDM

Leptin (LEP) is a hormone secreted by the adipose tissue of an organism, and its content in the serum is proportional to the proportion of animal adipose tissue [28]. In a normal body, its function is to maintain energy homeostasis [29]. LEP is mainly combined with leptin receptor (LEPR), also known as obesity receptor (ob-R), to play a biological effect. ob-R includes 6 subtypes spliced by diabetes gene (db gene) selective RNA splicing enzyme. ob-R can be divided into three categories: (1) Long ob-Rb, which is expressed in all immune cells, also highly expressed in the hypothalamus; (2) Short ob-Rc/d, which binds JAK kinase and activates the JAK-STAT pathway, is associated with LEP degradation; (3) Soluble ob-Re helps to maintain the normal level of LEP, thus limiting the biological action of cytokines. LEP can effectively inhibit appetite and induce energy consumption, mainly through the hypothalamic arcuate nucleus neurons to control weight and energy balance. In the bodies of normal pregnant women, the level of LEP in the early pregnancy is 2–3 times higher than that in the non-pregnancy period, and gradually increases [30]. The peak appears in late pregnancy, and the LEP level decreases sharply after delivery [31]. A study showed that the LEP level of GDM patients in early pregnancy was higher than that of normal pregnant women [32], but the ratio of third/first trimester was lower than that of normal pregnant women. This may indicate that GDM patients have impaired compensatory capacity for LEP. Maternal pre-pregnancy BMI is related to the hypomethylation status of leptin promoter in maternal blood samples [33]. Maternal LEP levels were not related to diet or exercise control [34]. This may be one of the reasons for the poor therapeutic effect of diet control in individual pregnant women with GDM. In addition, placenta is the main production site of leptin during pregnancy and is independent of the regulation of adipose tissue [35]. Leptin and leptin receptor were reported to be overexpressed in the placenta of GDM compared to the placenta of healthy pregnancy [36]. LEP can also induce the production of Human chorionic gonadotropin (HCG) in trophoblastic cells, and HCG, as a placental growth hormone, is involved in the regulation of glycolipid metabolism. However, leptin is a large protein molecule that cannot pass through the placental barrier, so it is independent of the maternal blood LEP and forms a separate regulatory mechanism. The placental leptin levels in the one twin with small for gestational age (SGA) were significantly higher in comparison with the other normal weight twin of a dichorionic twin pregnancy [37]. This also suggests that leptin regulation in placenta is independent in the same maternal blood environment. When obesity occurs during pregnancy, leptin resistance occurs in the body. At this time, the maternal brain cannot receive the signal of leptin level increase, and the appetite is out of control, thus leading to insulin resistance and the occurrence of GDM; however, the regulation mechanism of leptin in placenta has not been reported. LEP can induce phosphorylation of vascular endothelial growth factor receptor (VEGFR2) in umbilical vein endothelial cells, thus promoting nutrient interaction [38]. Abnormal leptin levels at the end of pregnancy are positively correlated with neonatal obesity [39].

#### 3.1.2. Regulation of Leptin Axis

The maintenance of energy homeostasis by LEP depends on two main factors: food intake and energy consumption. When the normal body ingests food, the adipose tissue will secrete leptin, which will be sensed by a neural circuit called the melanocortin system located in the hypothalamus through the blood, and release the signal of ‘full’, thus inhibiting the appetite of people. This system consists of primary and secondary neurons that communicate with each other [40,41]. The primary neurons, proopiomelanocortin (POMC) neurons, express anorexic proopiomelanocortin (Pomc) neurons and cocaine and amphetamine regulatory transcript (Cart). The secondary neurons are hypothalamic neuropeptide Y/agouti-related peptide (Npy/AgRP) neurons, which express agouti-related peptide (AgRP) and neuropeptide Y (Npy). These two peptides have an appetite-stimulating effect [42] and antagonistic functions [43], with their somas located in the arcuate nucleus of the hypothalamus (Arc). Leptin signal transduction is mediated by the JAK2/STAT3 pathway [44]. Negative regulators of JAK2 include suppressor of cytokine signaling 3 (SOCS3), Protein Tyrosine Phosphatase Non Receptor Type 1(PTPN1), also known as PTP1B. The up regulation of SOCS3 in POMC neurons leads to the damage of STAT3 signal, resulting in leptin resistance and obesity [45]. PTP1B deficient mice are allergic to leptin and resistant to diet-induced obesity [46]. In addition to the JAK2/STAT3 pathway, body weight control involves α-MSH peptide activated melanocortin receptor 4 (MC4R) signaling pathway, which is transcripted by the POMC gene. MC4R is located on chromosome 18 of the genome, mainly expressed in the paraventricular nucleus (PVN) of the hypothalamus. The activation of MC4R pathway mainly inhibits the effects of orexin, ghrelin, neuropeptide Y and Agouti-related protein [47,48]. MC4R is also highly expressed in pancreatic tissue, and its expression level is closely related to the regulation of insulin secretion [49]. Pancreas islet MC4R may affect the expression of Proprotein Convertase Subtilisin/Kexin Type 1 (PC1/3) through cAMP and β-repressor 1 pathway to regulate Glucagon Like Peptide 1(GLP-1) and insulin secretion [50].

Leptin resistance is also associated with sympathetic nerve conduction of impaired adipose in obese individuals. Leptin resistance at the central level may block negative feedback from the anti-inflammatory effects of the Sympathetic nervous system (SNS). Leptin resistance can lead to increased levels of inflammation in the body. For example, leptin can shift immune cells from Th1 to Th17 by increasing levels of Interferon-γ (IFN-γ).

#### 3.1.3. Methylation Changes of the Leptin Axis in GDM Patients

**Figure 3:**
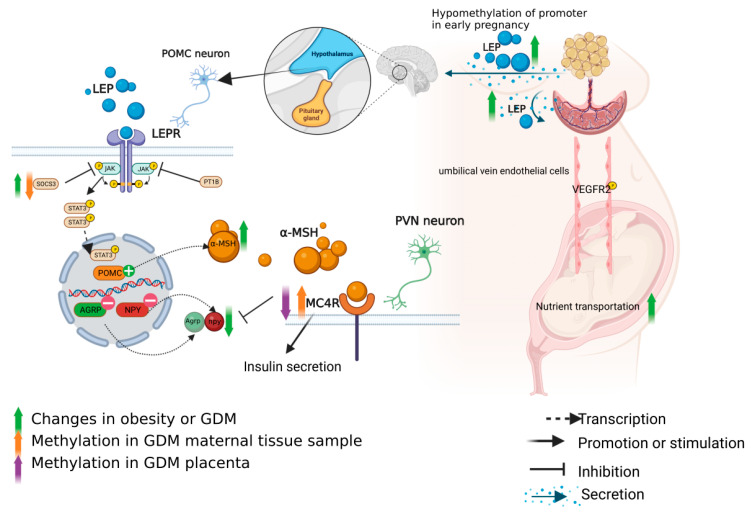
Leptin signal transduction and DNA methylation changes of related molecules in GDM and obesity. The images in this figure were taken from https://biorender.com/ (accessed on 22 November 2022).

Compared with normal pregnant women, the placental leptin promoter methylation level of GDM was increased [51], but leptin and leptin receptor were overexpressed in the placenta of GDM patients [36]. Under normal circumstances, the hypermethylation of a promoter will lead to the decrease of the expression of corresponding genes. This phenomenon cannot be explained simply by this theory. There may be more important factors that increase the expression of leptin, and DNA methylation is only one of the regulatory mechanisms. In addition, the level of MC4R DNA methylation in placentas of GDM women is lower than that of normal pregnant women. In the 2 h oral glucose tolerance test (OGTT), the DNA methylation level on the maternal side at CpG1 was positively correlated with the glucose concentration [52]. However, methylation sites in the placenta showed hypomethylation and increased mRNA expression, revealing a completely different methylation direction from that of the maternal side. It can be speculated that the maternal side tried to compensate for the increase of leptin secretion from the placenta, but the final result could not be completely offset by the subsequent result of leptin secretion from the placenta. In addition, the mRNA expression of SOCS3 in visceral fat of GDM women increased, and there was no significant difference in promoter methylation [53]. The methylation level of the SOCS3 promoter region in saliva of obese pregnant women was lower than that of the control group [54]. The increase of SOCS3 expression leads to the down-regulation of JAK2 pathway and the impairment of STAT3 signal, leading to leptin resistance, obesity and abnormal glucose metabolism [41] (Figure 3). In conclusion, placental leptin, MC4R and DNA methylation of SOCS3 in maternal samples play a certain role in the pathogenesis of GDM. However, the pathway of leptin on the maternal side is very complex, and the regulatory mechanism of leptin in the placenta remains unclear, so further studies are needed to clarify it.

### 3.2. Function of Adiponectin in GDM and Methylation Changes

Adiponectin (ADIPOQ) is an adipokine secreted by the adipose tissue, which plays a crucial role in maintaining insulin sensitivity, glucose and lipid homeostasis [55]. The low adiponectin level in early pregnancy is a risk marker for the onset of GDM [56]. Adiponectin works by activating the AMPK, mTOR, PI3K/Akt, MAPK, PPAR-α, STAT3 and NF kB pathways, among which the most important is the AMPK pathway which promotes the utilization of glucose through increasing fatty acid oxidation, glucose uptake at skeletal muscle level and decreasing gluconeogenesis in liver [57]. In animal models, adiponectin can enhance insulin sensitivity [58]. Hypoadiponectinemia can be observed in maternal blood of GDM [59]. Adiponectin expression was observed in the placental syncytiotrophoblast, and significantly down-regulated adiponectin mRNA was also observed in the placenta of GDM patients [60]. TNF-α and other proinflammatory mediators can inhibit adiponectin transcription in adipocytes [61]. The level of TNF-α in serum of GDM patients is higher than that of normal pregnant women, which may explain the low level of serum adiponectin. However, how adiponectin participates in the blood glucose regulation of GDM has not been revealed. Previous studies have shown that there is interaction between adiponectin, AdipoR1, IGF-I, IGF-IR and estrogen receptor α(ER-α) in breast cancer [62]. Similar hormonal interactions between the placenta and breast cancer may also lead to macrosomia, but further research is needed to prove this hypothesis. The study on methylation found that the methylation of adiponectin related genes may indeed participate in their respective gene regulation, but it was only slightly changed in GDM patients. However, the methylation of adiponectin DNA in cord blood was significantly changed in affected offspring [63]. The offspring of GDM women showed increased adiponectin DNA methylation and decreased adiponectin gene expression in SAT (subcutaneous fat). There was no significant difference in the plasma adiponectin methylation level [64].

### 3.3. Role of Chemerin in GDM and Methylation Changes

Chemerin is a newly discovered adipokine. Its mRNA is expressed in human placenta, especially in stromal cells and extravillous trophoblast cells, but its level is lower than that of adipose tissue and liver. When decidualization occurs during placental formation, the level of chemerin increases, which may affect the accumulation of NK cells and vascular remodeling in early pregnancy [65]. Studies have shown that the level of chemerin in blood and milk of GDM women is higher than that of normal pregnant women. However, in the comparison of chemerin concentration in serum, placenta, subcutaneous and visceral adipose tissue between GDM women and obese and non-obese healthy women [66], chemerin levels among healthy non-obese women, GDM women and healthy obese women are significantly different, which also indicates that the level of chemerin is closely related to BMI. Compared with normal mothers and their infants, GDM mothers and their newborns have a higher ratio of unmethylated chemerin genes in cord blood [67]. This finding shows that methylation regulation has a certain role in GDM. As a newly discovered adipokine, chemerin should be investigated regarding its pathway and expression regulation.

## 4. GDM Insulin Resistance Related Mechanisms and Methylation Changes

### 4.1. Molecular Mechanism of GDM Insulin Resistance

Insulin resistance is the main direct cause of GDM. One of the functions of insulin is to transport dietary glucose to the organs that need and store energy, such as the liver, skeletal muscle and adipose tissue. An important glucose transporter, glucose transporter-4 (GLUT4), is a major transporter that leads to the use of glucose into cells as energy. Compared to a normal pregnancy, GDM patients have a reduced basal and maximum insulin stimulating transport rates (per surface area) by 38 and 60%, respectively [68], thus leading to insulin resistance. Although the abundance of insulin receptors is generally unaffected, reduced tyrosine or increased serine/threonine phosphorylation of insulin receptors (IR) can inhibit insulin signaling [69]. In addition, the expression and phosphorylation of insulin signaling downstream regulators in GDM change in terms of insulin receptor substrate-1 (IRS1), insulin receptor substrate 2 (IRS2), Phosphatidylinositol-3-kinase subunit p85 (PIK3R1), and Phosphoinositol-3-kinase-α (PIK3CA). These transcription factors play an important role in insulin metabolism. Among the insulin receptor substrate family, IRS1 and IRS2 are the most commonly expressed, and they can bind the PI3K (especially the p85 subunit) signaling protein [70]. PIK3R1 (regulatory subunit) and PIK3CA (catalytic subunit) need to be combined to function as an enzyme [71] that stimulates the downstream AKT/mTORC signaling pathway to promote glucose metabolism, cell proliferation and survival. p101 (encoded by the PIK3R5 gene) and the p87 subunit formed heterodimerization complexes with p110γ, which were mainly regulated by the G protein coupled receptor GPCR, and also activated the AKT/mTORC signaling pathway to exert biological effects [72]. Failure of insulin signaling results in inadequate translocation of the plasma membrane of glucose transporter 4 (GLUT4), leading to insulin resistance, as shown in Figure 2.

### 4.2. Methylation Changes of Insulin Resistance in GDM

Studies have shown that the expressions of PIK3CA, PIK3R1, IRS1 and IRS2 in visceral fat are significantly reduced in the key downstream signaling pathway of IR in GDM patients, but are not related to promoter DNA methylation [73]. Another study found that differential methylation of the PIK3R5 gene was detected in the maternal blood of GDM patients in early pregnancy, with the expression of hypermethylation (β > 0) [74], suggesting that the change of its methylation had an impact on the maternal PI3K/AKT/mTOR signaling pathway. However, whether it is related to the pathogenesis of GDM has not been verified in the current research. In vitro experiments have found that Calmodulin Binding Activator 1 (CAMTA1) can slow the secretion of insulin under high blood glucose. [75]. Compared with normal pregnant women, the GDM group showed different methylation patterns of DNA methylation of CAMTA1 gene CpG [76]. Thus, insulin speed may be regulated by the level of CAMTA1 methylation.

## 5. Changes and Roles of Cytokines in GDM and Their Methylation Changes

### 5.1. TNF-α

The occurrence of GDM in pregnant women is correlated with cytokines. Cytokine changes may be the underlying pathophysiological mechanism of insulin resistance during pregnancy. The importance of low-grade inflammatory states in the pathogenesis of insulin resistance has recently become apparent. Proinflammatory cytokines have been found to both impair insulin signaling and inhibit insulin release from beta cells. Previous studies have shown that elevated tumor necrosis factor-α (TNF-α) is the most important independent predictor of insulin sensitivity [77]. In GDM patients, the level of TNF-α in blood was higher in the second and third trimesters than that in normal pregnant women [78]. The placental tissue of GDM patients releases more TNF-α in vitro under high glucose conditions [79]. TNF-α negatively regulates the PI3K/AKT signaling pathway through serine phosphorylation of Insulin Receptor Substrate 1, as shown in Figure 2, and reduces glucose transport of GLUT-4, thereby reducing glucose entry into cells and resulting in insulin resistance [80]. In addition, TNF-α and other pro-inflammatory mediators can inhibit the transcription of adiponectin in adipocytes [61], thereby over activating the insulin /IGF-1 pathway. Studies have shown that the mRNA expression of TNF-α in visceral fat of GDM patients is increased, and that the promoter methylation level is lower than that of normal pregnant women [53]. This suggests that methylation may be an important factor for the increase in TNF-α mRNA level in GDM mothers. However, there is no study on TNF-α methylation in placenta. To sum up, the underlying cause of TNF-α increase is still unknown. In addition to maternal factors, the placenta produces more TNF-α because it is in a high-glycemic environment. Therefore, it cannot be ruled out that the high level of TNF-α in GDM maternal blood is partly due to the translocation of the plasma through the placental barrier after placenta secretion to the maternal blood. Hence, the placenta may also play a key role in TNF-α in the pathogenesis of GDM.

### 5.2. IL-10

Interleukin-10 (IL-10) is an important anti-inflammatory cytokine, which can be synthesized and secreted by many kinds of cells. It can inhibit a variety of inflammatory cells to produce inflammatory cytokines. IL-10 not only plays an important regulatory role in the immune response of the body, but also plays a broader role in many tissues and organs, including participating in cell activation, proliferation and differentiation [81]. It has been reported that the plasma IL-10 level of GDM patients is significantly lower than that of the healthy control group. The low IL-10 level is negatively related to excessive insulin resistance of GDM patients, indicating that low IL-10 may increase insulin resistance of GDM patients [82,83]. The average IL-10 methylation level of GDM patients was significantly reduced compared with that of the maternal blood of the control group [84].

## 6. Conclusions

**Figure 4:**
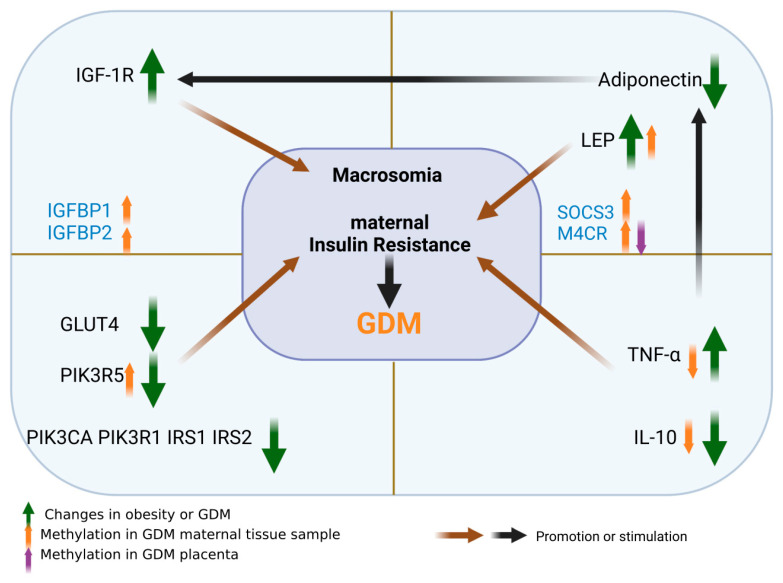
Role of various molecules and their methylation in the pathogenesis of GDM. The images in this figure were taken from https://biorender.com/ (accessed on 22 November 2022).

The presence of placenta delicately changes the secretion of hormones in pregnant women and the types of substance that antagonize insulin increases. In addition, leptin resistance in obese patients leads to excessive energy intake and affects the secretion of insulin, thus forming a microenvironment of hyperglycemia at the maternal–fetal interface. Therefore, the body and the maternal–fetal interface are in a state of slight inflammation and TNF-α increases. As a result, the level of maternal adiponectin is decreased, which further leads to the inhibition of insulin/IGF/AKT axis signal transduction, and then to the decrease in insulin stimulation glucose transport efficiency of GLU-4, aggravating insulin resistance. Methylation changes of many key molecules, such as IGFBP-1, IGFBP-2, MC4R, SOCS3, chemerin, CAMTA1, PIK3R5 and TNF-α, which play an important role in the pathogenesis of GDM, as shown in Figure 4. Hormone levels such as leptin and IGF-1 sharply decrease after the delivery of the placenta, and quickly recover to the level of non-pregnancy, which also shows the powerful role of the placenta as an endocrine organ and even overpowers the maternal health to provide adequate nutrition for the fetus. However, there are still many unknown factors in the regulation of placenta on hormones and its influence on maternal organism and fetus, which warrant further studies. The limitations of sample selection and methylated cell types have become obstacles to a thorough study of the etiology of GDM. In the future, the maturity of single cell sequencing technology and the emergence of single cell methylation sequencing technology will help us to deepen our understanding of the role of maternal and placental DNA methylation changes in the pathogenesis of gestational diabetes mellitus.

## Figures and Tables

**Table 1:** Compared with normal pregnant women, the peripheral blood changes of obese pregnant women may lead to GDM. The peripheral blood of GDM patients was also compared.

Category	Name	Maternal Obesity	GDM
Cytokines	TNF-α	↑ or →	↑
	IL-6	↑	↑
Adipokines	Leptin	↑	↑
	Adiponectin	↓	↓
Immune cells	M1 macrophage	↑	↑ or →
	NK cells	↑	cytotoxic and cytokine-secreting cells ↑
	Th1 and Tc lymphocytes	↑	↑
	Tregs	↓	↑

↑ represents the increase of peripheral blood level, ↓ represents the decrease of peripheral blood level, and → represents no significant change of peripheral blood level.

## Data Availability

The datasets used during the current study are available from the corresponding author on reasonable request.

## References

[1] American Diabetes Association (2004). Gestational diabetes mellitus. Diabetes Care.

[2] Cho N.H., Shaw J.E., Karuranga S., Huang Y., da Rocha Fernandes J.D., Ohlrogge A.W., Malanda B. (2018). IDF Diabetes Atlas: Global estimates of diabetes prevalence for 2017 and projections for 2045. Diabetes Res. Clin. Pract..

[3] Werner E.F., Has P., Tarabulsi G., Lee J., Satin A. (2016). Early postpartum glucose testing in women with gestational diabetes mellitus. Am. J. Perinatol..

[4] Ziller M.J., Gu H., Müller F., Donaghey J., Tsai L.T., Kohlbacher O., De Jager P.L., Rosen E.D., Bennett D.A., Bernstein B.E. (2013). Charting a dynamic DNA methylation landscape of the human genome. Nature.

[5] Gutierrez-Arcelus M., Ongen H., Lappalainen T., Montgomery S.B., Buil A., Yurovsky A., Bryois J., Padioleau I., Romano L., Planchon A. (2015). Tissue-specific effects of genetic and epigenetic variation on gene regulation and splicing. PLoS Genet..

[6] Luo Z.C., Nuyt A.M., Delvin E., Audibert F., Girard I., Shatenstein B., Cloutier A., Cousineau J., Djemli A., Deal C. (2012). Maternal and fetal IGF-I and IGF-II levels, fetal growth, and gestational diabetes. J. Clin. Endocrinol. Metab..

[7] Grissa O., Yessoufou A., Mrisak I., Hichami A., Amoussou-Guenou D., Grissa A., Djrolo F., Moutairou K., Miled A., Khairi H. (2010). Growth factor concentrations and their placental mRNA expression are modulated in gestational diabetes mellitus: Possible interactions with macrosomia. BMC Pregnancy Childbirth.

[8] Halle M., Berg A., Northoff H., Keul J. (1998). Importance of TNF-alpha and leptin in obesity and insulin resistance: A hypothesis on the impact of physical exercise. Exerc. Immunol. Rev..

[9] Lee M.H., Jeon Y.J., Lee S.M., Park M.H., Jung S.C., Kim Y.J. (2010). Placental gene expression is related to glucose metabolism and fetal cord blood levels of insulin and insulin-like growth factors in intrauterine growth restriction. Early Hum. Dev..

[10] McIntyre H.D., Zeck W., Russell A. (2009). Placental growth hormone, fetal growth and the IGF axis in normal and diabetic pregnancy. Curr. Diabetes Rev..

[11] Langer O., Yogev Y., Most O., Xenakis E.M. (2005). Gestational diabetes: The consequences of not treating. Am. J. Obstet. Gynecol..

[12] Valsamakis G., Kumar S., Creatsas G., Mastorakos G. (2010). The effects of adipose tissue and adipocytokines in human pregnancy. Ann. N. Y. Acad. Sci..

[13] Martín-Estal I., Castilla-Cortázar I., Castorena-Torres F. (2021). The placenta as a target for alcohol during pregnancy: The close relation with IGFs signaling pathway. Rev. Physiol. Biochem. Pharmacol..

[14] Kavran J.M., McCabe J.M., Byrne P.O., Connacher M.K., Wang Z., Ramek A., Sarabipour S., Shan Y., Shaw D.E., Hristova K. (2014). How IGF-1 activates its receptor. eLife.

[15] Moltke I., Grarup N., Jørgensen M.E., Bjerregaard P., Treebak J.T., Fumagalli M., Korneliussen T.S., Andersen M.A., Nielsen T.S., Krarup N.T. (2014). A common Greenlandic TBC1D4 variant confers muscle insulin resistance and type 2 diabetes. Nature.

[16] Karlsson H.K., Zierath J.R., Kane S., Krook A., Lienhard G.E., Wallberg-Henriksson H. (2005). Insulin-stimulated phosphorylation of the Akt substrate AS160 is impaired in skeletal muscle of type 2 diabetic subjects. Diabetes.

[17] Hwa V., Oh Y., Rosenfeld R.G. (1999). The insulin-like growth factor-binding protein (IGFBP) superfamily. Endocr. Rev..

[18] Jones J.I., Clemmons D.R. (1995). Insulin-like growth factors and their binding proteins: Biological actions. Endocr. Rev..

[19] Shang M., Wen Z. (2018). Increased placental IGF-1/mTOR activity in macrosomia born to women with gestational diabetes. Diabetes Res. Clin. Pract..

[20] Yoon M.S. (2017). The role of mammalian target of rapamycin (mTOR) in insulin signaling. Nutrients.

[21] Cheng S.C., Quintin J., Cramer R.A., Shepardson K.M., Saeed S., Kumar V., Giamarellos-Bourboulis E.J., Martens J.H., Rao N.A., Aghajanirefah A. (2014). mTOR- and HIF-1α-mediated aerobic glycolysis as metabolic basis for trained immunity. Science.

[22] Steyn A., Crowther N.J., Norris S.A., Rabionet R., Estivill X., Ramsay M. (2019). Epigenetic modification of the pentose phosphate pathway and the IGF-axis in women with gestational diabetes mellitus. Epigenomics.

[23] Pantham P., Aye I.L., Powell T.L. (2015). Inflammation in maternal obesity and gestational diabetes mellitus. Placenta.

[24] Šimják P., Cinkajzlová A., Anderlová K., Pařízek A., Mráz M., Kršek M., Haluzík M. (2018). The role of obesity and adipose tissue dysfunction in gestational diabetes mellitus. J. Endocrinol..

[25] De Luccia T.P.B., Pendeloski K.P.T., Ono E., Mattar R., Pares D.B.S., Yazaki Sun S., Daher S. (2020). Unveiling the pathophysiology of gestational diabetes: Studies on local and peripheral immune cells. Scand. J. Immunol..

[26] Zhang Y., Proenca R., Maffei M., Barone M., Leopold L., Friedman J.M. (1994). Positional cloning of the mouse obese gene and its human homologue. Nature.

[27] Kralisch S., Bluher M., Paschke R., Stumvoll M., Fasshauer M. (2007). Adipokines and adipocyte targets in the future management of obesity and the metabolic syndrome. Mini Rev. Med. Chem..

[28] Lisboa P.C., Oliveira E., Fagundes A.T., Santos-Silva A.P., Conceição E.P., Passos M.C., Moura E.G. (2012). Postnatal low protein diet programs leptin signaling in the hypothalamic-pituitary-thyroid axis and pituitary TSH response to leptin in adult male rats. Horm. Metab. Res. Horm. Stoffwechselforsch. Horm. Metab..

[29] D’Souza A.M., Neumann U.H., Glavas M.M., Kieffer T.J. (2017). The glucoregulatory actions of leptin. Mol. Metab..

[30] Plows J.F., Stanley J.L., Baker P.N., Reynolds C.M., Vickers M.H. (2018). The pathophysiology of gestational diabetes mellitus. Int. J. Mol. Sci..

[31] Pérez-Pérez A., Toro A., Vilariño-García T., Maymó J., Guadix P., Dueñas J.L., Fernández-Sánchez M., Varone C., Sánchez-Margalet V. (2018). Leptin action in normal and pathological pregnancies. J. Cell. Mol. Med..

[32] Powe C.E., Huston Presley L.P., Locascio J.J., Catalano P.M. (2019). Augmented insulin secretory response in early pregnancy. Diabetologia.

[33] Lesseur C., Armstrong D.A., Paquette A.G., Koestler D.C., Padbury J.F., Marsit C.J. (2013). Tissue-specific Leptin promoter DNA methylation is associated with maternal and infant perinatal factors. Mol. Cell. Endocrinol..

[34] Tessier D.R., Ferraro Z.M., Gruslin A. (2013). Role of leptin in pregnancy: Consequences of maternal obesity. Placenta.

[35] Kinalski M., Sledziewski A., Kowalska I., Telejko B., Kuźmicki M., Kretowski A., Majkowicz-Młynarczyk A., Kinalska I. (2004). Postpartum maternal plasma leptin levels and their relationship to gestational diabetes mellitus. Med. Wieku Rozw..

[36] Pérez-Pérez A., Maymó J.L., Gambino Y.P., Guadix P., Dueñas J.L., Varone C.L., Sánchez-Margalet V. (2013). Activated translation signaling in placenta from pregnant women with gestational diabetes mellitus: Possible role of leptin. Horm. Metab. Res. = Horm. Stoffwechselforsch. = Horm. Metab..

[37] Lewandowski K.C., Biesiada L., Grzesiak M., Sakowicz A. (2020). C-Peptide and leptin system in dichorionic, small and appropriate for gestational age twins-possible link to metabolic programming?. Nutr. Diabetes.

[38] Garonna E., Botham K.M., Birdsey G.M., Randi A.M., Gonzalez-Perez R.R., Wheeler-Jones C.P. (2011). Vascular endothelial growth factor receptor-2 couples cyclo-oxygenase-2 with pro-angiogenic actions of leptin on human endothelial cells. PLoS ONE.

[39] Catalano P.M., Presley L., Minium J., Hauguel-de Mouzon S. (2009). Fetuses of obese mothers develop insulin resistance in utero. Diabetes Care.

[40] van der Klaauw A.A. (2018). Neuropeptides in Obesity and Metabolic Disease. Clin. Chem..

[41] Maejima Y., Sakuma K., Santoso P., Gantulga D., Katsurada K., Ueta Y., Hiraoka Y., Nishimori K., Tanaka S., Shimomura K. (2014). Oxytocinergic circuit from paraventricular and supraoptic nuclei to arcuate POMC neurons in hypothalamus. FEBS Lett..

[42] Zhang L., Hernandez-Sanchez D., Herzog H. (2019). Regulation of feeding-related behaviors by arcuate neuropeptide y neurons. Endocrinology.

[43] Ollmann M.M., Wilson B.D., Yang Y.K., Kerns J.A., Chen Y., Gantz I., Barsh G.S. (1997). Antagonism of central melanocortin receptors in vitro and in vivo by agouti-related protein. Science.

[44] Zhou Y., Rui L. (2013). Leptin signaling and leptin resistance. Front. Med..

[45] Reed A.S., Unger E.K., Olofsson L.E., Piper M.L., Myers M.G., Xu A.W. (2010). Functional role of suppressor of cytokine signaling 3 upregulation in hypothalamic leptin resistance and long-term energy homeostasis. Diabetes.

[46] Bence K.K., Delibegovic M., Xue B., Gorgun C.Z., Hotamisligil G.S., Neel B.G., Kahn B.B. (2006). Neuronal PTP1B regulates body weight, adiposity and leptin action. Nat. Med..

[47] Farooqi I.S., O’Rahilly S. (2014). 20 years of leptin: Human disorders of leptin action. J. Endocrinol..

[48] Krishna R., Gumbiner B., Stevens C., Musser B., Mallick M., Suryawanshi S., Maganti L., Zhu H., Han T.H., Scherer L. (2009). Potent and selective agonism of the melanocortin receptor 4 with MK-0493 does not induce weight loss in obese human subjects: Energy intake predicts lack of weight loss efficacy. Clin. Pharmacol. Ther..

[49] Mansour M., White D., Wernette C., Dennis J., Tao Y.X., Collins R., Parker L., Morrison E. (2010). Pancreatic neuronal melanocortin-4 receptor modulates serum insulin levels independent of leptin receptor. Endocrine.

[50] Ni Z., Wang Y., Shi C., Zhang X., Gong H., Dong Y. (2022). Islet MC4R Regulates PC1/3 to Improve insulin secretion in T2DM mice via the cAMP and β-arrestin-1 pathways. Appl. Biochem. Biotechnol..

[51] Lesseur C., Armstrong D.A., Paquette A.G., Li Z., Padbury J.F., Marsit C.J. (2014). Maternal obesity and gestational diabetes are associated with placental leptin DNA methylation. Am. J. Obstet. Gynecol..

[52] Franzago M., Porreca A., D’Ardes M., Di Nicola M., Di Tizio L., Liberati M., Stuppia L., Vitacolonna E. (2022). The obesogenic environment: Epigenetic modifications in placental melanocortin 4 receptor gene connected to gestational diabetes and smoking. Front. Nutr..

[53] Rancourt R.C., Ott R., Ziska T., Schellong K., Melchior K., Henrich W., Plagemann A. (2020). Visceral adipose tissue inflammatory factors (TNF-Alpha, SOCS3) in gestational diabetes (GDM): Epigenetics as a clue in GDM pathophysiology. Int. J. Mol. Sci..

[54] Mandò C., Abati S., Anelli G.M., Favero C., Serati A., Dioni L., Zambon M., Albetti B., Bollati V., Cetin I. (2022). Epigenetic profiling in the saliva of obese pregnant women. Nutrients.

[55] Brochu-Gaudreau K., Rehfeldt C., Blouin R., Bordignon V., Murphy B.D., Palin M.F. (2010). Adiponectin action from head to toe. Endocrine.

[56] Bao W., Baecker A., Song Y., Kiely M., Liu S., Zhang C. (2015). Adipokine levels during the first or early second trimester of pregnancy and subsequent risk of gestational diabetes mellitus: A systematic review. Metab. Clin. Exp..

[57] Dalamaga M., Diakopoulos K.N., Mantzoros C.S. (2012). The role of adiponectin in cancer: A review of current evidence. Endocr. Rev..

[58] Berg A.H., Combs T.P., Du X., Brownlee M., Scherer P.E. (2001). The adipocyte-secreted protein Acrp30 enhances hepatic insulin action. Nat. Med..

[59] Fasshauer M., Blüher M., Stumvoll M. (2014). Adipokines in gestational diabetes. Lancet Diabetes Endocrinol..

[60] Chen J., Tan B., Karteris E., Zervou S., Digby J., Hillhouse E.W., Vatish M., Randeva H.S. (2006). Secretion of adiponectin by human placenta: Differential modulation of adiponectin and its receptors by cytokines. Diabetologia.

[61] Bruun J.M., Lihn A.S., Verdich C., Pedersen S.B., Toubro S., Astrup A., Richelsen B. (2003). Regulation of adiponectin by adipose tissue-derived cytokines: In vivo and in vitro investigations in humans. Am. J. Physiol. Endocrinol. Metab..

[62] Mauro L., Naimo G.D., Ricchio E., Panno M.L., Andò S. (2015). Cross-talk between adiponectin and IGF-IR in breast cancer. Front. Oncol..

[63] Ott R., Stupin J.H., Melchior K., Schellong K., Ziska T., Dudenhausen J.W., Henrich W., Rancourt R.C., Plagemann A. (2018). Alterations of adiponectin gene expression and DNA methylation in adipose tissues and blood cells are associated with gestational diabetes and neonatal outcome. Clin. Epigenet..

[64] Houshmand-Oeregaard A., Hansen N.S., Hjort L., Kelstrup L., Broholm C., Mathiesen E.R., Clausen T.D., Damm P., Vaag A. (2017). Differential adipokine DNA methylation and gene expression in subcutaneous adipose tissue from adult offspring of women with diabetes in pregnancy. Clin. Epigenet..

[65] Estienne A., Bongrani A., Reverchon M., Ramé C., Ducluzeau P.H., Froment P., Dupont J. (2019). Involvement of novel adipokines, chemerin, visfatin, resistin and apelin in reproductive functions in normal and pathological conditions in humans and animal models. Int. J. Mol. Sci..

[66] Tsiotra P.C., Halvatsiotis P., Patsouras K., Maratou E., Salamalekis G., Raptis S.A., Dimitriadis G., Boutati E. (2018). Circulating adipokines and mRNA expression in adipose tissue and the placenta in women with gestational diabetes mellitus. Peptides.

[67] Fatima S.S., Rehman R., Muhammad J.S., Martins R., Mohammed N., Khan U. (2022). Association of chemerin gene promoter methylation in maternal blood and breast milk during gestational diabetes. J. Dev. Orig. Health Dis..

[68] Garvey W.T., Maianu L., Zhu J.H., Hancock J.A., Golichowski A.M. (1993). Multiple defects in the adipocyte glucose transport system cause cellular insulin resistance in gestational diabetes. Heterogeneity in the number and a novel abnormality in subcellular localization of GLUT4 glucose transporters. Diabetes.

[69] Copps K.D., White M.F. (2012). Regulation of insulin sensitivity by serine/threonine phosphorylation of insulin receptor substrate proteins IRS1 and IRS2. Diabetologia.

[70] McCurdy C.E., Klemm D.J. (2013). Adipose tissue insulin sensitivity and macrophage recruitment: Does PI3K pick the pathway?. Adipocyte.

[71] Metz H.E., Houghton A.M. (2011). Insulin receptor substrate regulation of phosphoinositide 3-kinase. Clin. Cancer Res. Off. J. Am. Assoc. Cancer Res..

[72] Roskoski R. (2021). Properties of FDA-approved small molecule phosphatidylinositol 3-kinase inhibitors prescribed for the treatment of malignancies. Pharmacol. Res..

[73] Rancourt R.C., Ott R., Schellong K., Melchior K., Ziska T., Henrich W., Plagemann A. (2019). Visceral adipose tissue alteration of PI3KR1 expression is associated with gestational diabetes but not promoter DNA methylation. Adipocyte.

[74] Wu P., Farrell W.E., Haworth K.E., Emes R.D., Kitchen M.O., Glossop J.R., Hanna F.W., Fryer A.A. (2018). Maternal genome-wide DNA methylation profiling in gestational diabetes shows distinctive disease-associated changes relative to matched healthy pregnancies. Epigenetics.

[75] Mollet I.G., Malm H.A., Wendt A., Orho-Melander M., Eliasson L. (2016). Integrator of stress responses calmodulin binding transcription activator 1 (Camta1) regulates miR-212/miR-132 expression and insulin secretion. J. Biol. Chem..

[76] Dias S., Adam S., Rheeder P., Louw J., Pheiffer C. (2019). Altered genome-wide DNA methylation in peripheral blood of South African women with gestational diabetes mellitus. Int. J. Mol. Sci..

[77] Kirwan J.P., Hauguel-De Mouzon S., Lepercq J., Challier J.C., Huston-Presley L., Friedman J.E., Kalhan S.C., Catalano P.M. (2002). TNF-alpha is a predictor of insulin resistance in human pregnancy. Diabetes.

[78] Dong Y., Chauhan M., Betancourt A., Belfort M., Yallampalli C. (2018). Adipose tissue inflammation and adrenomedullin overexpression contribute to lipid dysregulation in diabetic pregnancies. J. Clin. Endocrinol. Metab..

[79] Coughlan M.T., Oliva K., Georgiou H.M., Permezel J.M., Rice G.E. (2001). Glucose-induced release of tumour necrosis factor-alpha from human placental and adipose tissues in gestational diabetes mellitus. Diabet. Med. J. Br. Diabet. Assoc..

[80] Peraldi P., Spiegelman B. (1998). TNF-alpha and insulin resistance: Summary and future prospects. Mol. Cell. Biochem..

[81] Laffer B., Bauer D., Wasmuth S., Busch M., Jalilvand T.V., Thanos S., Meyer Zu Hörste G., Loser K., Langmann T., Heiligenhaus A. (2019). Loss of IL-10 promotes differentiation of microglia to a M1 phenotype. Front. Cell. Neurosci..

[82] Charles B.A., Doumatey A., Huang H., Zhou J., Chen G., Shriner D., Adeyemo A., Rotimi C.N. (2011). The roles of IL-6, IL-10, and IL-1RA in obesity and insulin resistance in African-Americans. J. Clin. Endocrinol. Metab..

[83] Yang Y., Liu L., Liu B., Li Q., Wang Z., Fan S., Wang H., Wang L. (2018). Functional defects of regulatory T Cell through interleukin 10 mediated mechanism in the induction of gestational diabetes mellitus. DNA Cell Biol..

[84] Kang J., Lee C.N., Li H.Y., Hsu K.H., Wang S.H., Lin S.Y. (2018). Association of interleukin-10 methylation levels with gestational diabetes in a Taiwanese population. Front. Genet..

